# High‐throughput collagen fingerprinting of intact microfaunal remains; a low‐cost method for distinguishing between murine rodent bones

**DOI:** 10.1002/rcm.7483

**Published:** 2016-03-07

**Authors:** Mike Buckley, Muxin Gu, Sanu Shameer, Soyab Patel, Andrew T. Chamberlain

**Affiliations:** ^1^ Faculty of Life SciencesManchester Institute of Biotechnology131 Princess StreetManchesterM1 7DNUK

## Abstract

**Rationale:**

Microfaunal skeletal remains can be sensitive indicators of the contemporary ecosystem in which they are sampled and are often recovered in owl pellets in large numbers. Species identification of these remains can be obtained using a range of morphological criteria established for particular skeletal elements, but typically dominated by a reliance on cranial characters. However, this can induce biases under different environmental and taphonomic conditions. The aim of this research was to develop a high‐throughput method of objectively identifying rodent remains from archaeological deposits using collagen fingerprinting, most notably the identification of rats from other myomorph rodents as a means to identify disturbances in the archaeofauna through the presence of invasive taxa not contemporary with the archaeological deposits.

**Methods:**

Collagen was extracted from complete microfaunal skeletal remains in such a manner as to leave the bones morphologically intact (i.e., weaker concentration of acid than previously used over shorter length of time). Acid‐soluble collagen was then ultrafiltered into ammonium bicarbonate and digested with trypsin prior to dilution in the MALDI matrix and acquisition of peptide mass fingerprints using a matrix‐assisted laser desorption/ionisation time‐of‐flight (MALDI‐TOF) mass spectrometer.

**Results:**

Collagen fingerprinting was able to distinguish between *Rattus*, *Mus*, *Apodemus* and *Micromys* at the genus level; at the species level, *R. rattus* and *R. norvegicus* could be separated whereas *A. flavicollis* and *A. sylvaticus* could not. A total of 12,317 archaeological microvertebrate samples were screened for myomorph signatures but none were found to be invasive rats (*Rattus*) or mice (*Mus*). Of the contemporary murine fauna, no harvest mice (*Micromys*) were identified and only 24 field mouse (*Apodemus*) discovered.

**Conclusions:**

As a result, no evidence of recent bioturbation could be inferred from the faunal remains of these archaeological deposits. More importantly this work presents a method for high‐throughput screening of specific taxa and is the first application of collagen fingerprinting to microfaunal remains of archaeological specimens. © 2016 The Authors. *Rapid Communications in Mass Spectrometry* Published by John Wiley & Sons Ltd.

## Microfaunal remains

The most abundant organic remains on archaeological sites are vertebrate skeletal remains or fragments thereof. During the last half century, with improving recovery strategies, such zooarchaeological assemblages are increasingly dominated by overwhelming numbers of microvertebrate remains, including small mammals, small birds and reptiles, amphibians and fish.^1^ Although the majority of these species are not usually considered to have been directly hunted in large numbers by past human populations, with the exception of fish, most are considered potential palaeoenvironmental indicators (e.g.^2^). Although reptiles and amphibians are more appropriate proxies for inferences to temperature, and studied for their potential as early warning indicators for over‐grazing in modern arid climates,^3^, ^4^ mammals have the advantage that they are generally more tolerant of slight changes in climate^5^ and therefore more likely to establish sufficient population sizes and enter the archaeological record. On islands such as Britain, mammals have been considered valuable palaeoenvironmental indicators particularly in prehistory,^6^ due to the periodic connection of Britain with the continent during the Quaternary Period. In some cases they have been used as a tool for mammalian biostratigraphy, specifically being used to identify the presence of multiple post‐Anglian interglacial periods prior to the Last (Ipswichian) interglacial.^7^ Small mammal remains have also been shown to be indicative of different agricultural cycles^8^ and the wider human impact on the environment in historic times, particularly with respect to the introduction of invasive species.^9^

## Cave microfauna

With regards to the use of faunal remains for palaeoenvironmental inferences, British Pleistocene vertebrate assemblages are typically recovered from cave and fissure, marine, fluviatile and lacustrine sites.^10^ Of these, cave sites usually offer by far the richest accumulations of skeletal material because they offer ideal conditions for bone preservation (i.e., relatively constant temperature in comparison to open sites). However, cave faunal accumulations occur via several processes: some are dominated by non‐biological/environmental accumulations of remains, such as allochthonous material transported in by streams or in some cases whereby the cave acts as a pitfall trap, but most assemblages are the prey‐remains of various avian and terrestrial carnivores such as owls, hyaenas, foxes, wolves and humans. Whereas the larger carnivores, such as hyaena, may indiscriminately drag large prey into the cave, small vertebrate assemblages accumulated by owls roosting in cave roofs may not only be biased by the environment, but also by the dietary preferences of the species of owl responsible.^10^, ^11^, ^12^

However, one of the greatest threats to the stratigraphic integrity of archaeological cave deposits is that of disturbed stratigraphy caused by burrowing animals especially rodents, lagomorphs and large mustelids^13^, ^14^ – one of the most common occurrences in caves being caused by the brown rat. Faunal turbation of deposits has the potential to introduce the remains of stratigraphically younger animals into older deposits.

## Murine rodents

This research focuses specifically on the three most widely introduced taxa worldwide, focusing on the brown (*Rattus norvegicus*) and black rats (*R. rattus*) and including the house mouse (*Mus musculus*). It also investigates the murine rodents most likely encountered in palaeolithic sites of Britain, the wood mouse (*Apodemus sylvaticus*) and the yellow‐necked field mouse (*A. flavicollis*) as well as the Eurasian harvest mouse (*Micromys minutus*), the latter of which were present early in the Holocene ~10 Ka,^15^ but it is uncertain how much earlier in the Pleistocene it arrived.^16^ Although these rodents can usually be readily separated on morphological grounds with cranial elements, post‐cranial remains are much less widely identified,^11^ despite many elements being more robust than cranial bones.

The earliest invasive rodent to enter Britain via commensalism with humans was the house mouse, which became associated with humans in western Asia^17^ but arrived in Britain by the Iron Age (~1000 BC^18^). Both black and brown rats are believed to have originated in eastern Asia, with the former originating in the Indo‐Malayan region^19^ and the latter further north on the plains of northern China and Mongolia.^20^ Although both species, through commensalism, travelled across Europe and into Britain with humans, the black rat arrived much earlier, with remains being recovered from mid‐third century AD London^21^ and fifth century AD York.^22^ Brown rats are thought to have reached Britain much later, introduced by trading ships by the late 1720s, but it is these rodents that are more likely to cause disruption through bioturbation within archaeological sites.

## Species identification

However, even though many of the microfaunal remains recovered from archaeological assemblages are often relatively intact compared with the remains of larger faunal remains, the morphology of most post‐cranial skeletal elements between some distinct species are so similar as to make separation difficult.^23^, ^24^ As a result, it is often only the cranial remains (and most commonly teeth, often with optical magnification) that are used for such assessments (e.g.^11^), despite the potential bias that is likely present towards some taxa over others (i.e., those with less robust mandibular and maxillary bone structure are likely to be under‐represented).

Although biomolecular methods to species identification, both DNA‐ and protein‐based methods, have been proposed for fragmentary taxa,^25^, ^26^, ^27^ they have not been considered more appropriate than morphological approaches of intact remains. DNA‐based methods of species identification offer population‐level information (e.g.^28^), but with variable DNA preservation in owl pellet faunas^29^ and resultant financial costs far too high to be feasible with large sample sizes.

Current protein‐based methods are typically one or two orders of magnitude less expensive but are equally limited in scope, and whereby analysis costs for thousands of samples would currently be too high for the returned information. There are two forms of protein analysis most typically used for species identification of fragmentary bone, one being based on immunological reactivity,^26^ the other using soft‐ionization mass spectrometry (i.e., proteomics‐based methodologies) including fingerprinting (e.g.^25^) and liquid chromatography (LC)‐based peptide sequencing approaches.^30^ This research seeks to translate a recently developed method of species identification by collagen fingerprinting into a high‐throughput technique capable of analysing thousands of samples at relatively low cost and within a short period of time. In application to the study of micromammals, this first case study is applied to the Upper Palaeolithic site of Pin Hole Cave, Derbyshire, UK, specifically targeting the murine rodents potentially present within the thousands of vertebrate remains recovered to date.

## Case study: Pin Hole Cave, Creswell Crags, UK

Pin Hole Cave (SK533742) is one of several archaeologically important caves at the limestone gorge of Creswell Crags (Derbyshire/Nottinghamshire border), UK, that were occupied by humans in at least three distinct phases during the last ice age from ~50–10 Ka.^31^, ^32^ The first human inhabitants were the Neanderthals (50–60 Ka), followed by a Gravettian occupation (between ~40–28 Ka), and then once more during the Magdalenian (~14–12 Ka), with apparent hyaena denning in the interim periods. One of these caves, Church Hole Cave, is known for having the northernmost cave art in Europe.^33^ Some of these other caves, such as at Mother Grundy*'*s Parlour, yielded remains of much older fauna, such as hippopotami, dating back to the Last Interglacial warm period (~125 Ka^34^).

The caves have been excavated several times since the nineteeenth century, and Pin Hole was the first at Creswell to be excavated, but it was done so on several occasions, the last being in the 1980s. Being formed in Magnesian limestone, the cave measures 31 m long with an approximate width of only 1–2 m wide and a small side chamber at approximately 17 m into the main passage. The first excavations in 1894 only went 5 m into the cave where they met flowstone. The bulk of the currently excavated material was collected in the 1920s, with over 5 m sediments including a lower cave earth with interbedded stalagmite floor dated to around 110 Ka, and an upper cave earth that is capped by a breccia dated to less than 19 Ka.^31^, ^32^ However, these excavations left several metres of deposit remaining at the back of the cave, a section of which was later excavated in the 1980s using more modern techniques of sample recovery and recording, collecting ~30,000 archaeological finds from the top metre of deposit alone, some of the bones of which form the focus of this investigation. Although both hyaena and human inhabitants are known to have collected some of the faunal remains, the majority of remains were of microfauna, likely the remains of owls roosting in the cave roof.

The aims of the current research are to present a high‐throughput biomolecular methodology to the targeted species identification of thousands of microfaunal remains, focusing on the identification of murine rodent remains.

## Experimental

Collagen extractions from modern reference samples were carried out similar to methods described by van der Sluis *et al*.^35^ using overnight demineralisation in 0.5 mL 0.6 M hydrochloric acid (HCl) and buffer exchanged into 50 mM ammonium bicarbonate (ABC; twice with 0.5 mL, collected with 0.1 mL) using single 30 kDa molecular weight cut‐off (MWCO) ultrafilter units. Following ultrafiltration, overnight tryptic digestion (0.2 μg sequencing‐grade trypsin; Promega, UK) was carried out at 37°C, diluted in 0.1% trifluoroacetic acid (TFA) and spotted onto a 384‐well stainless steel Bruker Ultraflex target plate with an equal volume of 10 mg/mL α‐cyanohydroxycinnamic acid matrix. Each plate was calibrated with multiple spots containing the using five peptides; Angiotensin II, bradykinin fragment 1‐7, P_14_R, ACTH fragment 18‐39 and insulin chain B (Sigma‐Aldrich MSCAL2). A total of 2000 laser acquisitions were obtained per spot using a Bruker Ultraflex II matrix assisted laser desorption/ionisation (MALDI) time of flight (TOF) mass spectrometer. However, due to the structural damage caused by our standard approach to the archaeological test specimens (Pin Hole Cave specimens from Manchester Museum), for the analyses of intact archaeological remains, the HCl concentration was reduced to 0.3 M and only used over a 4 h demineralisation period following which most archaeological specimens remained morphologically intact. The 12,307 Pin Hole Cave archaeological bone specimens from the 1980s excavations were loaned from the Creswell Crags Museum and Heritage Centre collections and translocated into 96‐well microtitre plates. Then 0.5 mL of 0.3 M HCl was added and removed to 30 kDa MWCO 96‐well ultrafilter units. Following centrifugation at 3700 rpm, the flow‐through was discarded and 0.5 mL 50 mM ABC was added and centrifuged as above. This step was repeated once more and then 200 μL added to the filters, mixed and removed to separate plates; half of this was removed and digested as described above. Following digestion, 2 μL samples were spotted onto 384‐well stainless steel Bruker Ultraflex target plates following dilution in 10 mg/mL α‐cyanohydroxycinnamic acid matrix and allowed to dry. MALDI analyses were carried out on calibrated plates as described above, where spectra from archaeological protein digests were considered of good enough standard for further investigation when more than 10 peaks of *m*/*z* >2000 with a signal/noise ratio of >3 were observed.

To assist with peptide interpretation, the brown rat tryptic digest was also analysed by LC/MS/MS (Waters nanoAcquity UPLC system coupled to a Thermo Scientific Orbitrap Elite mass spectrometer) on which the peptides were concentrated on a pre‐column (20 mm × 180 μm) then separated on a 1.7 μM Waters nanoAcquity BEH (Ethylene Bridged Hybrid) C18 analytical column (75 mm × 250 μm i.d.), using a gradient from 99% buffer A (0.1% formic acid (FA) in H_2_O)/1% buffer B (0.1% FA in ACN) to 25% B in 45 min at 200 nL min^–1^. Peptides were selected for fragmentation automatically by data‐dependent analysis. Proteomics data files were searched using Mascot v2.2.06 (Matrix Science) against the publicly available SwissProt database.^36^ Standard searches were carried out using two missed cleavages, error tolerances of 0.5 *m*/*z* units (MS and MS/MS) and variable oxidation of methionine and hydroxylation of proline and lysine and deamidation of asparagines and glutamine modifications.

## Results

### Taxonomic resolution

**Figure 1:**
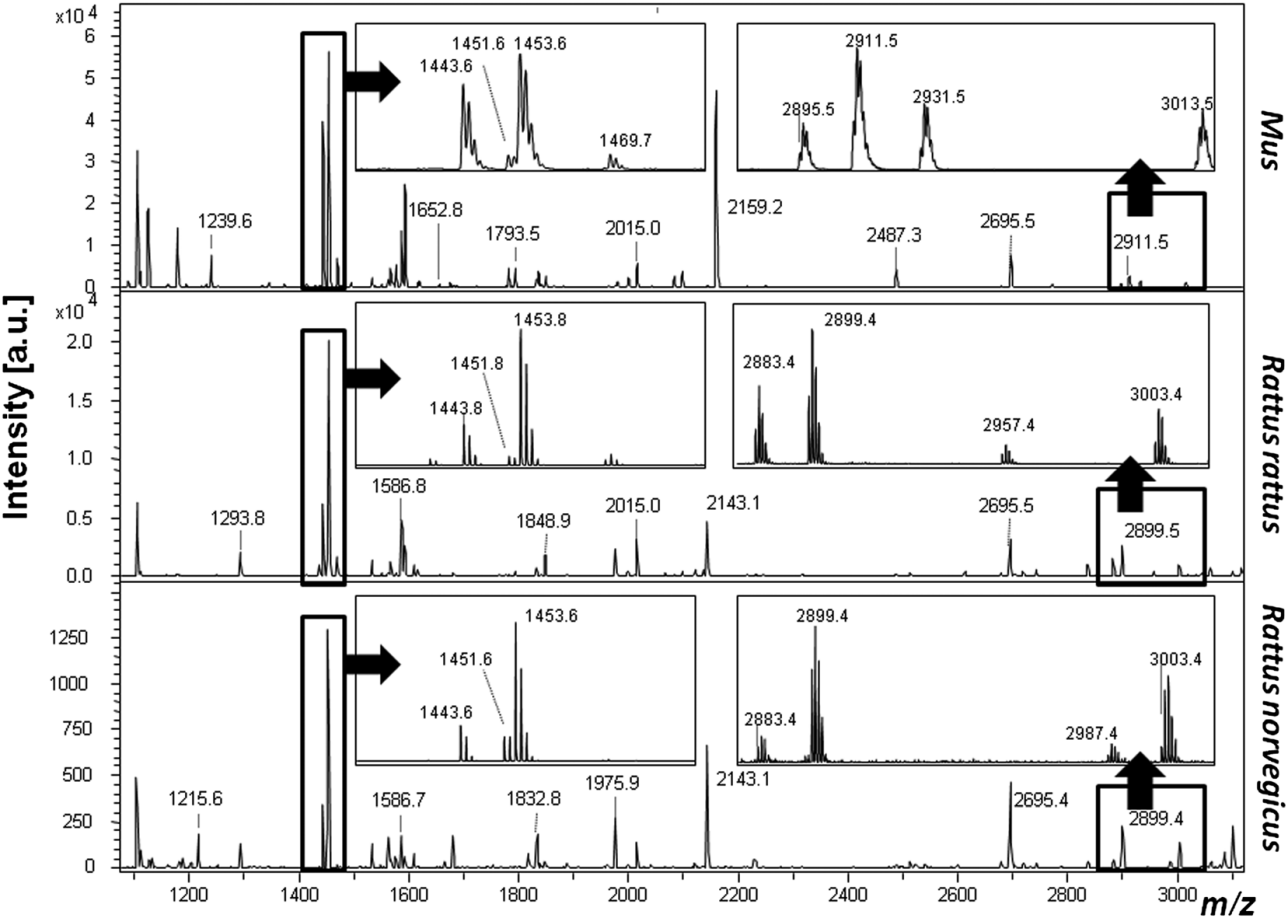
MALDI‐TOF mass spectra of tryptic digests of bone collagen from *Rattus* and *Mus*.

**Figure 2:**
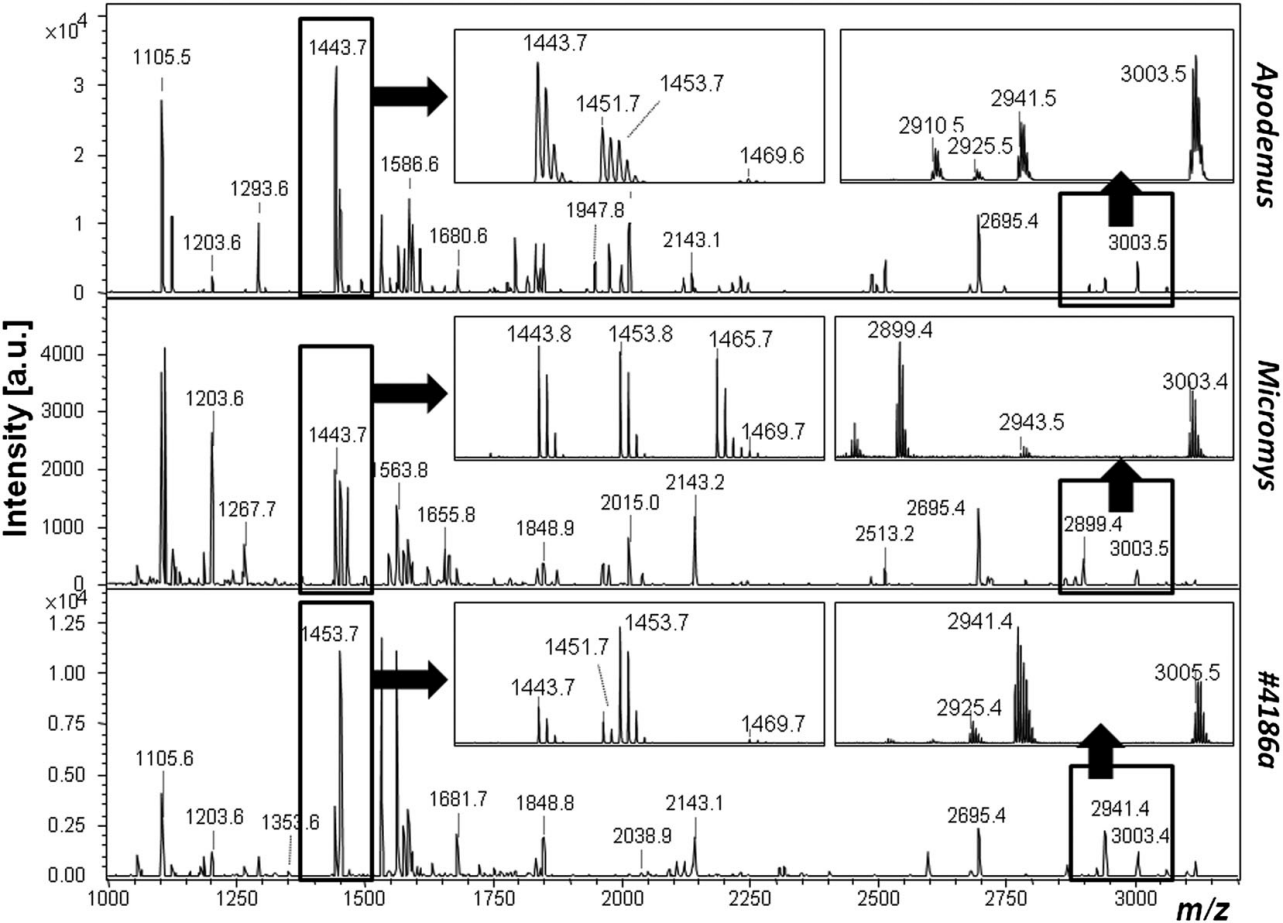
MALDI‐TOF mass spectra of tryptic digests of bone collagen from *Apodemus* and *Micromys* reference material as well as one example of *Apodemus* identified in the archaeological material.

Collagen fingerprints were obtained from modern identified specimens of *R. norvegicus*, *R. rattus*, *Mus musculus*, *Micromys minutus*, *A. sylvaticus* and *A. flavicollis* (Figs. 1, 2). Homologous species‐specific markers were observed for *Rattus* at *m*/*z* 2987/2957 (Fig. 1), but none could be identified that readily separated the two *Apodemus* species (*A. sylvaticus* and *A. flavicollis*). However, the peptide peak at *m*/*z* 1443.7 (representing the COL1A1 peptide GAAGPPGATGFPGAAGR as determined by LC/Orbitrap sequencing of the *R. norvegicus* specimen – see Supplementary Table S1, Supporting Information; underlined residue indicates hydroxylation site), homologous to an otherwise highly conserved marker typically at *m*/*z* 1459.7 in most vertebrates (representing GSAGPPGATGFPGAAGR; e.g.^30^, ^37^), appears specific to the myomorph rodents in this study, whereas the peptide marker at *m*/*z* 1451.7 (representing COL1A1 peptide GEPGPSGLPGPPGER; Supplementary Table S1) appears specific to the lineage within murine rodents that excludes the Eurasian harvest mouse (*Micromys minutus*). It is also noticeable that the peptide marker at *m*/*z* 2695.4 (representing the COL1A1 peptide GFSGLQGPPGSPGSPGEQGPSGASGPAGPR; Supplementary Table S1), which is highly conserved in other vertebrate taxa at *m*/*z* 2705.4 (GFSGLQGPPGPPGSPGEQGPSGASGPAGPR), is observed throughout these myomorph rodents.

### Archaeological results

Only 24 of the 7069 good‐quality spectra contained the murine markers identified in Table 1 (listed in Table 2). Closer inspection of these (i.e., the remainder of the peaks in each spectra) allowed for the identification of each as deriving from *Apodemus* spp. Only one of these had a morphological identification to species level (*Apodemus sylvaticus*; PH6368), with most of the others being indeterminate to at least the order level, i.e., ‘Rodentia’, ‘non‐bat’, or simply ‘indeterminate microvertebrate’ (Table 2). Five of these remains come from the same location in the cave deposits, four of which are potentially from the same individual as they were recovered initially as one find. Seven other distinct locations were also represented implying that these remains were accumulated at relatively distinct phases of the occupation of the cave site.

**Table 1:** Collagen peptide markers for rodents within this study

**Species**	**1 (A)**	**2**	**3**	**4 (B)**	**5 (D)**	**6**	**7 (F)**	**8 (G)**	**9**
*R. norvegicus*	1203.7	1443.7	1451.7	1453.7	2143.1	2695.4	2899.5	2987.5	3003.5
*R. rattus*	1203.7	1443.7	1451.7	1453.7	2143.1	2695.4	2899.5	2957.5	3003.5
*Apodemus*	1203.7	1443.7	1451.7	1453.7	2143.1	2695.4	2910.5	2941.5	3003.5
*Micromys*	1203.7	1443.7	1465.7	1453.7	2143.1	2695.4	2899.5	2943.5	3003.5
*Mus*	1194.7	1443.7	1451.7	1453.7	2159.1	2695.4	2911.5	2931.5	3013.5

Letters in brackets indicate markers identified for *Mus* and *Rattus* previously described.^25^

**Table 2:** Accession information relating to the only myomorph rodents identified by collagen fingerprinting in this study including Pin Hole (PH) accession code, original morphological identification and the location of the 10 cm^2^ square that the find was recovered from in terms of its westing, northing and depth from a datum point set at the start of the excavations

**PH code**	**Original ID**	**Location (Westing/Northing/Depth)**
15596a	‘Rodentia’	W14/N4/d126
14514	‘Rodentia’	W15/N6/d125
9232	‘Rodentia’	W15/N5/d115
2963	‘Rodentia’	W13/N3/d109
6368	*Apodemus sylvaticus*	W8/N1/flowstone
4826	Indeterminate microvertebrate	W7/N9/flowstone
4845c	‘Rodentia’	W6/N9/d32
4845d	‘Rodentia’	W6/N9/d32
4522	‘Rodentia’	W5/N9/d35
4186a	Indeterminate microvertebrate	W7/N2/d53
4429	‘Rodentia’	W13/N2/d94
4036a	Indeterminate microvertebrate (‘not bat’)	W3/N8/d36
4036b	Indeterminate microvertebrate (‘not bat’)	W3/N8/d36
4036c	Indeterminate microvertebrate (‘not bat’)	W3/N8/d36
4036d	Indeterminate microvertebrate (‘not bat’)	W3/N8/d36
6578b	Indeterminate	W15/N5/d109
	Mammal	
2904c	‘Rodentia’	W8/N1/flowstone
2904i	‘Rodentia’	W8/N1/flowstone
2904j	‘Rodentia’	W8/N1/flowstone
2904n	‘Rodentia’	W8/N1/flowstone
2904p	‘Rodentia’	W8/N1/flowstone
2837	‘Rodentia’	W15/N5/d107
2782	Indeterminate flake	W4/N14/d28
2789	‘Rodentia’	W5/N11/d29

## Discussion

### Taxonomic resolution of collagen fingerprinting within murine rodents

It is well established that the true rodents had evolved by the end of the Paleocene ~54 Ma in Asia,^38^ with the murid family (including myomorph rodents, hamsters, voles and gerbils) appearing by the end of the Eocene ~34 Ma. The major groups within this study, leading to *Apodemus*, *Micromys*, *Mus* and *Rattus*, all diverged from each other ~7–12 Ma;^39^, ^40^, ^41^ although the phylogeny of *Micromys* remains unclear, it is more frequently placed as a close relative to *Apodemus*.^42^ During the Pliocene, these rodent groups radiated widely, with apparent speciation within the already established genus *Apodemus* ~5.5 Ma^40^ and the genus *Rattus* first emerging ~3.5 Ma^43^, ^44^ with a native range predominantly in Asia.^45^ The black rat (*R. rattus*) is estimated to have diverged from the brown rat (*R. norvegicus*) ~2 Ma.^41^

The taxonomic resolution obtainable from the collagen fingerprints of the rodents within this study is not wholly consistent with what would be expected for a molecular clock. Particularly noticeable is the ability to discriminate within the *Rattus* genus, but not within the *Apodemus* genus, with the former having almost half the divergence time. This is likely simply related to the very low number of amino acid substitutions observed throughout, and perhaps also a bias in the partial nature of the collagen fingerprint, which itself only typically presents ~60–80 peptides representing ~40–50% of the collagen (I) alpha 1 and alpha 2 chain sequences available.^46^ However, apparent absences of such markers could also relate to the biochemical properties of each analyte, whereby particular amino acid substitutions could make the peptide less amenable to analysis and observation (e.g.^47^, ^48^, ^49^). Nonetheless, the ability to obtain genus‐level information in most,^25^, ^50^ and species‐level information in some mammals,^51^, ^52^ provides much greater levels of information than may be obtained with most non‐molecular methods in the absence of specific skeletal remains. In particular, *Apodemus sylvaticus* and *A. flavicollis* remains are known to be difficult to distinguish in most cases on morphology alone, particularly without intact crania.^53^

### Pin Hole Cave stratigraphy and assemblage accumulation

**Figure 3:**
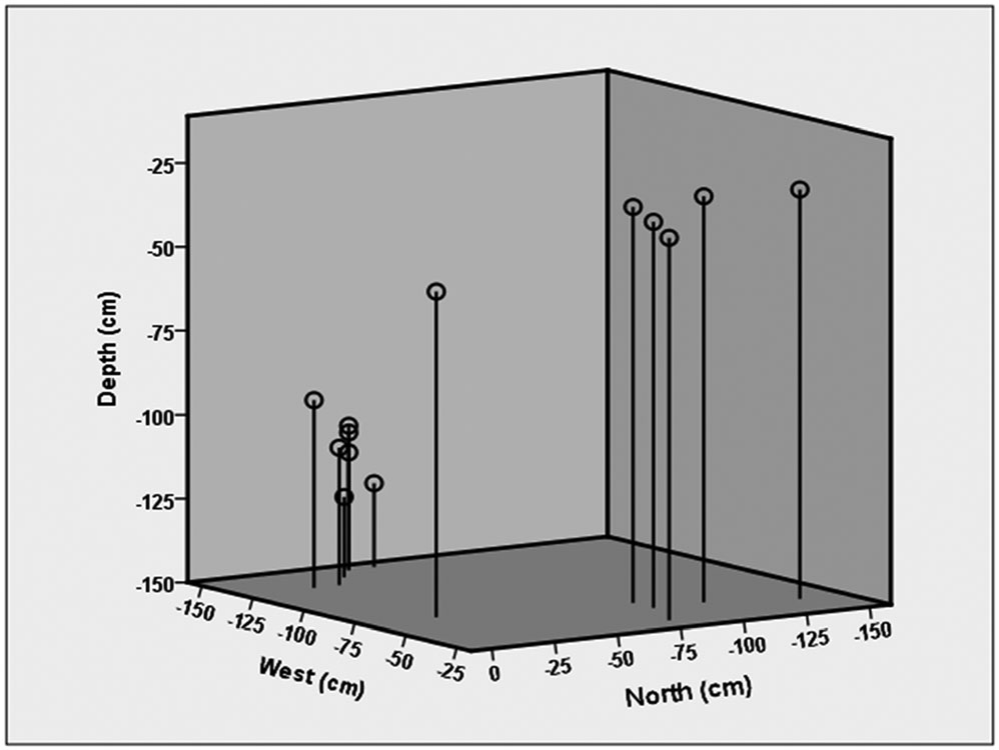
Spatial plot of the *Apodemus* remains from the cave deposit showing an incline that is consistent with the slope of the sediments described previously.

Due to the manner in which the Pin Hole Cave remains were collected, with the strategy to retain all ‘specimens’ that could potentially be important, many finds were retained in the bone archive that were clearly not bone but clumps of soil that immediately dissolved on contact with the HCl. This had a noticeable impact on the apparent success rate, which would appear to be only ~57% if all subsamples are assumed to originate from bone. Previous descriptions of the deposits within Pin Hole Cave, which are thought to have accumulated from an entrance in the ceiling at the rear of the cave, suggest a 36° slope of the sediments from north to south and east to west,^31^, ^32^ and therefore it is plausible that the *Apodemus* finds plotted on a similar incline in Fig. 3 are of a horizon with a temporal range much narrower than it would otherwise appear, perhaps during a single short phase of owl occupation of this cave.

Although it is expected that the majority of specimens derive from cricetid rodents, including numerous species of voles and lemmings, the aims of the present study were to present a targeted high‐throughput methodology for collagen fingerprinting in order to identify potential intrusive murine taxa as a means to assess the integrity of the stratigraphy. The findings that no remains of ‘exogenous’ taxa, such as *Rattus* or *Mus*, are present is reassuring in showing that the sedimentary stratigraphy recovered from the 1980s excavation area in the back of Pin Hole Cave was relatively undisturbed by these potentially intrusive creatures. The apparent absence of *Micromys* is not surprising given that not only do they reflect a much lower relative proportion of barn owl prey in modern accumulations typically <10% of prey,^54^ they are not known to have been present in Britain prior to the Holocene. The exhaustive study presented here, despite only being on one assemblage, adds further support to their absence at least in this region of Britain. The small number of *Apodemus* remains in relation to the total microvertebrate remains is seemingly very low, but is likely a reflection of the local environment. These animals inhabit a mostly woodland environment, as well as grasslands. *A. flavicollis* is typically considered a forest mouse, always in sites with trees or at least with larger seeded plants such as bushes, whereas *A. sylvaticus* is mainly found outside the forests, usually nearer the edges and preferring shrub and grassy areas.^55^

The bone accumulator for the microvertebrate remains is considered to be a form of owl, perhaps barn owl.^31^ Owls are generally nocturnal, and capable of hunting a wide variety of microfauna^12^ including many small mammals, birds, frogs, reptiles and fish – the latter of which are also present in the Pin Hole Cave assemblage. The micromammals in this case would have been swallowed whole, with the bones being regurgitated into small pellets. However, the contents of owl pellets do not necessarily represent the contents of prey consumed, with reports of up to 60% of consumed prey missing from experimental studies.^11^ However, some owls, such as the Tawny owl, hunt such a wide range of prey that they can offer good environmental indicators, once the digestability of different prey taxa have been considered.

### Wider applications to microvertebrate studies

This research has focused on the development of a technique capable of processing much larger numbers of samples for species identification than previously attempted. Microfaunal assemblages are more frequently reaching sample sizes in the hundreds of thousands of finds with more recent excavations (e.g.^56^) and therefore the introduction of a technique that allows for the inclusions of significant portions of these datasets could have a substantial impact on the palaeoenvironmental inferences for the region. Although there is a slight decrease in the quality of the observed fingerprint when compared with earlier methods, it more than makes up for this in the increase in throughput, and is likely to be improved upon as the technique develops. The greatest hindrance to this fingerprinting approach is the current requirement for comparison to reference spectra, although the accuracy of the taxonomic hierarchy that appears to be observed (e.g.^25^) needs to be further evaluated. The proposal of species‐specific biomarkers for a given set of taxa is to be tested with further studies, as in the example of our original identification of the peptide marker that discriminated sheep and goat collagen,^57^ being later tested by targeted DNA methods.^58^ Only one of the 24 *Apodemus* specimens identified in this study was morphologically identified to species level (*A. sylvaticus*), beyond the capabilities of the proposed fingerprinting method. Given the environment, it would be unsurprising if all of the identified specimens belong to *A. sylvaticus* and some may derive from the same individual (e.g., the four bearing Accession Number 4036). However, it is not yet possible to confirm this with the current methods.

## Conclusions

The proposed methodology allows for the collagen fingerprinting methodology to become much more applicable to large zooarchaeological assemblages. The taxonomic limitations of the technique is an obvious factor that needs further investigation, but as a cheap method that can work on such a large scale, it overcomes some of the problems inherent in traditional DNA‐based methods in that no prior knowledge is required to obtain the molecular identification. The targeted approach used here has wide geographical applications in improving our understanding of the palaeogeography of particular taxa. Future developments in a wider range of vertebrate collagen fingerprints will allow for studies in changing palaeobiodiversity through time, with particular importance in relation to climate change and changing vertebrate ecosystems.

## Supporting information

Supporting info item

## References

[1] N. Branch , M. Canti , P. Clark , C. Turney . Environmental Archaeology: Theoretical and Practical Approaches. Edward Arnold, London, 2005.

[2] G. Cuenca‐Bescós , L. G. Straus , M. R. González Morales , J. C. García Pimienta . The reconstruction of past environments through small mammals: from the Mousterian to the Bronze Age in El Mirón Cave (Cantabria, Spain). J. Archaeol. Sci. 2009, 36, 947.

[3] J. L. Read . Experimental trial of Australian arid zone reptiles as early warning indicators of overgrazing by cattle. Austral Ecol. 2002, 27, 55.

[4] C. McAlpine , R. Fensham , D. Temple‐Smith . Biodiversity conservation and vegetation clearing in Queensland: principles and thresholds. The Rangeland J. 2002, 24, 36.

[5] R. J. Rowe , R. C. Terry . Small mammal responses to environmental change: integrating past and present dynamics. J. Mammal. 2014, 95, 1157.

[6] A. Lister . Mammalian fossils and Quaternary biostratigraphy. Quaternary Sci. Rev. 1992, 11, 329.

[7] D. C. Schreve . Differentiation of the British late Middle Pleistocene interglacials: the evidence from mammalian biostratigraphy. Quaternary Sci. Rev. 2001, 20, 1693.

[8] J.‐D. Vigne , H. ène Valladas . Small mammal fossil assemblages as indicators of environmental change in northern Corsica during the last 2500 years. J. Archaeol. Sci. 1996, 23, 199.

[9] S. Valenzuela , F. Poitevin , R. Cornette , A. Bournery , J. Nadal , J.‐D. Vigne . Evolving ecosystems: ecological data from an Iron Age small mammal accumulation at Alorda Park (Catalonia, Spain). J. Archaeol. Sci. 2009, 36, 1248.

[10] A. Stuart . Pleistocene history of the British vertebrate fauna. Biol. Rev. 1974, 49, 225.

[11] P. Andrews . Owls, Caves, and Fossils: Predation, Preservation and Accumulation of Small Mammal Bones in Caves, with an Analysis of the Pleistocene Cave Faunas from Westbury‐Sub‐Mendip, Somerset, UK. University of Chicago Press, Chicago, 1990.

[12] I. Taylor . Barn Owls: Predator‐Prey Relationships And Conservation. Cambridge University Press, Cambridge, 2004.

[13] K. D. Fowler , H. J. Greenfield , L. O. van Schalkwyk . The effects of burrowing activity on archaeological sites: Ndondondwane, South Africa. Geoarchaeology 2004, 19, 441.

[14] E. J. Reitz , M. Shackley . Environmental Archaeology. Springer, USA, 2012.

[15] W. I. Montgomery , J. Provan , A. M. McCabe , D. W. Yalden . Origin of British and Irish mammals: disparate post‐glacial colonisation and species introductions. Quaternary Sci. Rev. 2014, 98, 144.

[16] S. Harris . History, distribution, status and habitat requirements of the harvest mouse (*Micromys minutus*) in Britain. Mammal Rev. 1979, 9, 159.

[17] T. Cucchi , Z. E. Kovács , R. Berthon , A. Orth , F. Bonhomme , A. Evin , et al. On the trail of Neolithic mice and men towards Transcaucasia: zooarchaeological clues from Nakhchivan (Azerbaijan). Biol. J. Linnean Soc. 2013, 108, 917.

[18] T. Cucchi , J. D. Vigne , J. C. Auffray . First occurrence of the house mouse (*Mus musculus domesticus* Schwarz & Schwarz, 1943) in the Western Mediterranean: a zooarchaeological revision of subfossil occurrences. Biol. J. Linnean Soc. 2005, 84, 429.

[19] K. P. Aplin , H. Suzuki , A. A. Chinen , R. T. Chesser , J. Ten Have , S. C. Donnellan , et al. Multiple geographic origins of commensalism and complex dispersal history of black rats. PloS ONE 2011, 6, e26357. 22073158 10.1371/journal.pone.0026357 PMC3206810

[20] R. A. Gibbs , G. M. Weinstock , M. L. Metzker , D. M. Muzny , E. J. Sodergren , S. Scherer , et al. Genome sequence of the Brown Norway rat yields insights into mammalian evolution. Nature 2004, 428, 493. 15057822 10.1038/nature02426

[21] P. Armitage , B. West , K. Steedman . New evidence of Black Rat in Roman London. The London Archaeologist 1984, 4, 375.

[22] J. Rackham . *Rattus rattus*: the introduction of the black rat into Britain. Antiquity 1979, 53, 112. 11620121 10.1017/s0003598x00042319

[23] E. Matisoo‐Smith , J. S. Allen . Name that rat: molecular and morphological identification of Pacific rodent remains. Int. J. Osteoarchaeol. 2001, 11, 34.

[24] J. H. Robins , M. Hingston , E. Matisoo‐Smith , H. A. Ross . Identifying *Rattus* species using mitochondrial DNA. Mol. Ecol. Notes 2007, 7, 717.

[25] M. Buckley , M. Collins , J. Thomas‐Oates , J. C. Wilson . Species identification by analysis of bone collagen using matrix‐assisted laser desorption/ionisation time‐of‐flight mass spectrometry. Rapid Commun. Mass Spectrom. 2009, 23, 3843. 19899187 10.1002/rcm.4316

[26] J. M. Lowenstein , J. D. Reuther , D. G. Hood , G. Scheuenstuhl , S. C. Gerlach , D. H. Ubelaker . Identification of animal species by protein radioimmunoassay of bone fragments and bloodstained stone tools. Forensic Sci. Int. 2006, 159, 182. 16191470 10.1016/j.forsciint.2005.08.007

[27] P. Tozzo , E. Ponzano , E. Novelli , M. Onisto , L. Caenazzo . Discrimination between human and animal DNA: application of a duplex polymerase chain reaction to forensic identification. Am. J. Forensic Med. Pathol. 2011, 32, 180. 21263288 10.1097/PAF.0b013e31820c2bba

[28] C. J. Kolman , N. Tuross . Ancient DNA analysis of human populations. Am. J. Phys. Anthropol. 2000, 111, 5. 10618586 10.1002/(SICI)1096-8644(200001)111:1<5::AID-AJPA2>3.0.CO;2-3

[29] S. Guimaraes , Y. Fernandez‐Jaivo , E. Stoetzel , O. Gorge , E. A. Bennett , C. Denys , et al. Owl pellets: a wise DNA source for small mammal genetics. J. Zool. 2015, 1.

[30] M. Buckley . A molecular phylogeny of Plesiorycteropus reassigns the extinct mammalian order ‘Bibymalagasia’. PloS ONE 2013, 8, e59614. 23555726 10.1371/journal.pone.0059614 PMC3608660

[31] A. L. Armstrong . Excavations in the Pin Hole Cave, Creswell Crags, Derbyshire. Proc. Prehistoric Soc. East Anglia. 1932, 6, 330.

[32] R. M. Jacobi , P. J. Rowe , M. A. Gilmour , R. Gruen , T. C. Atkinson . Radiometric dating of the Middle Palaeolithic tool industry and associated fauna of Pin Hole Cave, Creswell Crags, England. J. Quaternary Sci. 1998, 13, 29.

[33] P. G. Bahn . Britain's oldest art: the Ice Age cave art of Creswell Crags. English Heritage, 2009.

[34] R. Jenkinson , D. D. Gilbertson , J. Collis , G. Britain . In the shadow of extinction: a quaternary archaeology and palaeoecology of the lake, fissures and smaller caves at Creswell Crags. SSSI, Sheffield, 1984.

[35] L. Van der Sluis , H. Hollund , M. Buckley , P. De Louw , K. Rijsdijk , H. Kars . Combining histology, stable isotope analysis and ZooMS collagen fingerprinting to investigate the taphonomic history and dietary behaviour of extinct giant tortoises from the Mare aux Songes deposit on Mauritius. Palaeogeography, Palaeoclimatology, Palaeoecology 2014, 416, 80.

[36] Available: http://www.ebi.ac.uk/uniprot.

[37] C. Wadsworth , M. Buckley . Proteome degradation in fossils: investigating the longevity of protein survival in ancient bone. Rapid Commun. Mass Spectrom. 2014, 28, 605. 24519823 10.1002/rcm.6821 PMC4282581

[38] J. Meng , A. R. Wyss , M. R. Dawson , R. Zhai . Primitive fossil rodent from Inner Mongolia and its implications for mammalian phylogeny. Nature 1994, 370, 134. 8022481 10.1038/370134a0

[39] X. Liu , F. Wei , M. Li , X. Jiang , Z. Feng , J. Hu . Molecular phylogeny and taxonomy of wood mice (genus Apodemus Kaup, 1829) based on complete mtDNA cytochrome b sequences, with emphasis on Chinese species. Mol. Phylogenetics Evolution 2004, 33, 1. 10.1016/j.ympev.2004.05.011 15324834

[40] J. Michaux , P. Chevret , M.‐G. Filippucci , M. Macholan . Phylogeny of the genus Apodemus with a special emphasis on the subgenus Sylvaemus using the nuclear IRBP gene and two mitochondrial markers: cytochrome b and 12S rRNA. Mol. Phylogenetics Evolution 2002, 23, 123. 10.1016/S1055-7903(02)00007-6 12069545

[41] O. Verneau , F. Catzeflis , A. V. Furano . Determining and dating recent rodent speciation events by using L1 (LINE‐1) retrotransposons. Proc. Natl. Acad. Sci. 1998, 95, 11284. 9736728 10.1073/pnas.95.19.11284 PMC21634

[42] Y. Martin , G. Gerlach , C. Schlötterer , A. Meyer . Molecular phylogeny of European muroid rodents based on complete cytochrome b sequences. Mol. Phylogenetics Evolution 2000, 16, 37. 10.1006/mpev.1999.0760 10877938

[43] J. H. Robins , P. A. McLenachan , M. J. Phillips , L. Craig , H. A. Ross , E. Matisoo‐Smith . Dating of divergences within the Rattus genus phylogeny using whole mitochondrial genomes. Mol. Phylogenetics Evolution 2008, 49, 460. 10.1016/j.ympev.2008.08.001 18725306

[44] K. C. Rowe , K. P. Aplin , P. R. Baverstock , C. Moritz . Recent and rapid speciation with limited morphological disparity in the genus Rattus. Systemat. Biol. 2011, 60, 188. 10.1093/sysbio/syq092 21239388

[45] K. P. Aplin , T. Chesser , J. t. Have . Evolutionary biology of the genus Rattus: profile of an archetypal rodent pest. ACIAR Monograph Ser. 2003, 96, 487.

[46] M. Buckley , N. Larkin , M. Collins . Mammoth and Mastodon collagen sequences; survival and utility. Geochim. Cosmochim. Acta 2011, 75, 2007.

[47] A. R. Dongre , J. L. Jones , Á. Somogyi , V. H. Wysocki . Influence of peptide composition, gas‐phase basicity, and chemical modification on fragmentation efficiency: Evidence for the mobile proton model. J. Am. Chem. Soc. 1996, 118, 8365.

[48] K. F. Medzihradszky , J. M. Campbell , M. A. Baldwin , A. M. Falick , P. Juhasz , M. L. Vestal , et al. The characteristics of peptide collision‐induced dissociation using a high‐performance MALDI‐TOF/TOF tandem mass spectrometer. Anal Chem. 2000, 72, 552. 10695141 10.1021/ac990809y

[49] R. S. Brown , J. J. Lennon . Sequence‐specific fragmentation of matrix‐assisted laser‐desorbed protein/peptide ions. Anal. Chem. 1995, 67, 3990. 8633762 10.1021/ac00117a027

[50] M. Buckley , M. J. Collins . Collagen survival and its use for species identification in Holocene‐lower Pleistocene bone fragments from British archaeological and paleontological sites. Antiqua 2011, 1, e1.

[51] M. Buckley , S. Fraser , J. Herman , N. Melton , J. Mulville , A. Pálsdóttir . Species identification of archaeological marine mammals using collagen fingerprinting. J. Archaeol. Sci. 2014, 41, 631.

[52] N. Rybczynski , J. C. Gosse , C. R. Harington , R. A. Wogelius , A. J. Hidy , M. Buckley . Mid‐Pliocene warm‐period deposits in the High Arctic yield insight into camel evolution. Nat. Commun. 2013, 4, 1550. 23462993 10.1038/ncomms2516 PMC3615376

[53] A. L. Ruprecht . Bats (Chiroptera) as constituents of the food of barn owls *Tyto alba* in Poland. Ibis 1979, 121, 489.

[54] D. E. Glue . Food of the barn owl in Britain and Ireland. Bird Study 1974, 21, 200.

[55] P. Angelstam , L. Hansson , S. Pehrsson . Distribution borders of field mice Apodemus: the importance of seed abundance and landscape composition. Oikos 1987, 123.

[56] J. M. Garvey . Preliminary zooarchaeological interpretations from Kutikina Cave, south‐west Tasmania. Aust. Aboriginal Stud. 2006, 57.

[57] M. Buckley , S. W. Kansa , S. Howard , S. Campbell , J. Thomas‐Oates , M. Collins . Distinguishing between archaeological sheep and goat bones using a single collagen peptide. J. Archaeol. Sci. 2010, 37, 13.

[58] M. G. Campana , T. Robinson , P. F. Campos , N. Tuross . Independent confirmation of a diagnostic sheep/goat peptide sequence through DNA analysis and further exploration of its taxonomic utility within the Bovidae. J. Archaeol. Sci. 2013, 40, 1421.

